# Guidelines for delineation of lymphatic clinical target volumes for high conformal radiotherapy: head and neck region

**DOI:** 10.1186/1748-717X-6-97

**Published:** 2011-08-19

**Authors:** Hilke Vorwerk, Clemens F Hess

**Affiliations:** 1 Radiotherapy and Radiooncology, University Hospital Göttingen, Robert-Koch-Str. 40, 37073 Göttingen, Germany; 2 Radiotherapy and Radiooncology, University Marburg, Baldingerstrasse, 35043 Marburg, Germany

## Abstract

The success of radiotherapy depends on the accurate delineation of the clinical target volume. The delineation of the lymph node regions has most impact, especially for tumors in the head and neck region. The purpose of this article was the development an atlas for the delineation of the clinical target volume for patients, who should receive radiotherapy for a tumor of the head and neck region. Literature was reviewed for localisations of the adjacent lymph node regions and their lymph drain in dependence of the tumor entity. On this basis the lymph node regions were contoured on transversal CT slices. The probability for involvement was reviewed and a recommendation for the delineation of the CTV was generated.

## Introduction

The major problem in radiation treatment with IMRT technique is the failure to select and delineate the target accurately, especially in patients with head and neck cancer, in which a high risk of subclinical nodal disease exists. CT-based investigation is not sufficient to detect metastases smaller than one centimetre in diameter [1]. Since the lymph node status is the most important prognostic factor in patients with squamous cell cancer in the head and neck region, and due to the limitation of clinical staging, other factors, like histopathologic examinations, may help to predict metastatic lymph node involvement [1-3].

The lymphatic migration of tumor cells is usually stepwise and occurs in a predictable manner [4-6]. Detailed anatomical knowledge of the lymphatic network associated with each area of the body is essential to define all the sides in which the presence of metastatic nodes should be investigated and to delineate on a morphological basis the optimal target volume to be treated by high conformal radiotherapy [5,7]. An optimization of radiation techniques to maximize local tumor control and to minimize side effects in radiotherapy of head and neck tumors requires proper definition and delineation guidelines for the clinical target volume (CTV). Most previous results are consensus guidelines from different physicians [2,8,9].

The purpose of this article was to define the lymphatic CTV for the radiation treatment on a CT based atlas for tumors of the head and neck region to have a principle recipe for the delineation for clinical use. This atlas displays the clinically relevant nodal stations and their correlation with normal lymphatic pathways on a set of CT images.

## General anatomy

**Figure 1:**
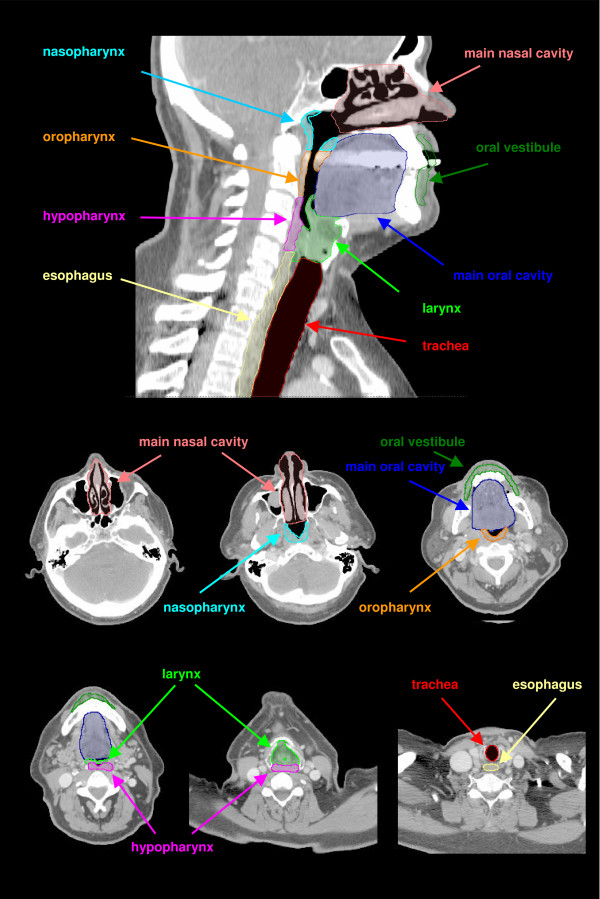
**Anatomic head and neck regions contoured on a sagittal DRR and transversal CT slices**.

The main nasal cavity includes the cavities of the interior nose between the vestibule of the nose and the Choana (Figure 1). The oral vestibule is located between the teeth and the lips and the cheek respectively. The alveolar process border the oral cavity lateral and ventral, whereas the velum and palatine border the oral cavity to the cranial side (Table 1). The caudal limit is the floor of the mouth. The pharynx is defined as the region of the combined respiratory and digestive system, which is located dorsal of the oral cavity and nasal cavity, incipient cranial at the skull base up to caudal at the beginning of the esophagus and the trachea. The pharynx is divided into three regions - nasopharynx, oropharynx and hypopharynx. The exact limits between these regions are not definitely defined. The nasopharynx is located at the cranial part of the pharynx and ends caudal at the velum palatinum. The nasopharynx includes the pharyngeal tonsil. The next section of the pharynx is the oropharynx, which ends at the top of the epiglottis. The third part of the pharynx is the hypopharynx, which begins cranial of the larynx and ends at the cranial ending of the cricoid cartilage behind the larynx. The larynx is subdivided into three parts: supraglottis, glottis and subglottis. The supraglottis is the vestibulum of the larynx, beginning at the entrance of the larynx down to the fissure between the plicae vestibulares. The glottis is the intermediate cavity between the rima vestibule and the glottis opening. The most caudal laryngeal region down to the entrance of the trachea is the subglottis (infraglottic cavity).

**Table 1:** Anatomic head and neck regions

anatomic region	description
nose and paranasal sinus	main nasal cavity
	vestibule of the nose
	maxillary sinus

oral cavity	gingiva
	hard palate
	buccal mucosa
	floor of the mouth
	ventral 2/3 of the tongue
	oral vestibule
	lips

salivary glands	parotid gland
	submandibular gland
	sublingual gland

nasopharynx	posterior wall of the pharynx beginning at the threshold between the soft and hard palatine up to the base of the skull
	nasal surface of the soft palatine
	palatine tonsil

oropharynx	pharyngeal tonsil
	arcus palatinus
	root of the tongue
	vallecula epiglottica
	posterior wall of the oropharynx
	oral surface of the soft palatine
	uvula

hypopharynx	posterior wall of the pharynx between the upper border of the epiglottis and the esophagus
	post cricoid region
	sinus piriformis

larynx	cricoid cartilage
	thyroid cartilage
	cartilages arytaenoideae
	epiglottis

## Lymph drainage

The lymphatic CTV encompasses pathologic lymph nodes with a safety margin and adjacent areas, which are at risk for tumor spread. Lymph nodes should be assessed as pathologic, if their diameter is more than 1 cm, all nodes with spherical rather than ellipsoidal shape, nodes containing inhomogeneities (suggestive of necrotic centers) or a cluster of three or more borderline nodes. In the node positive patients, an important factor to consider is the probability of capsular rupture and extracapsular extension. The lymphatic CTV do not only include lymph nodes (LN) with radiological criteria of involvement but also one or more adjacent lymph node regions [2,10,11]. The lymphatic drainage for each organ uses several pathways including the main collecting way, but also alternative routes [5]. These alternative routes should be included in the target volume definition in dependence of the feasibility for that route.

**Figure 2:**
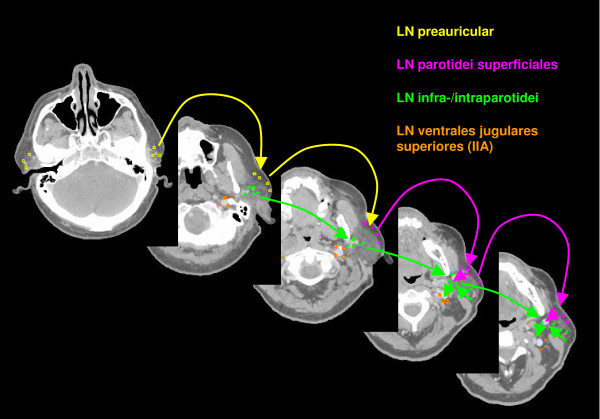
**Lymph regions and drain contoured in transversal CT slices: LN parotidei superficiales (pink) and LN parotidei profundi subdivided into LN preauriculares (yellow) and LN infra-/intraparotidei (light green) [1.8 cm slice thickness]**.

**Figure 3:**
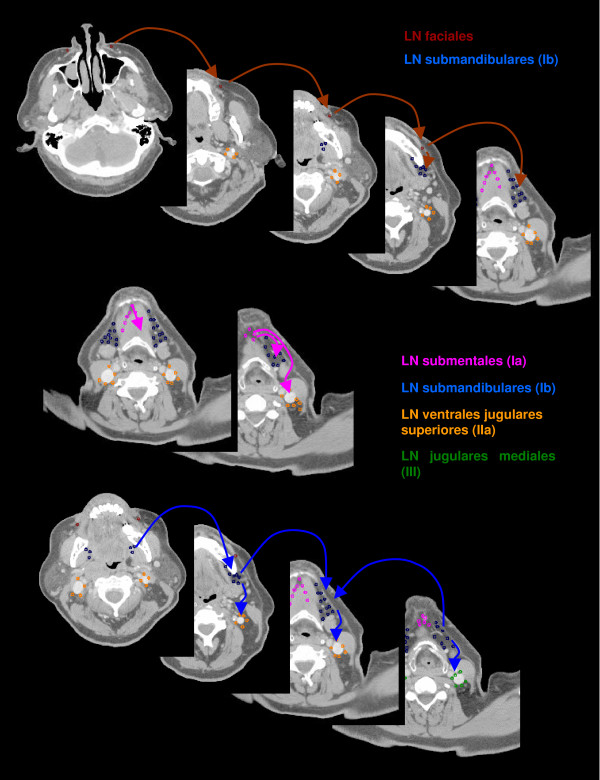
**Lymph regions and drain contoured in transversal CT slices: LN buccales (brown), LN submentales (pink) and LN submandibulares (dark blue) [1.8 cm slice thickness]**.

**Figure 4:**
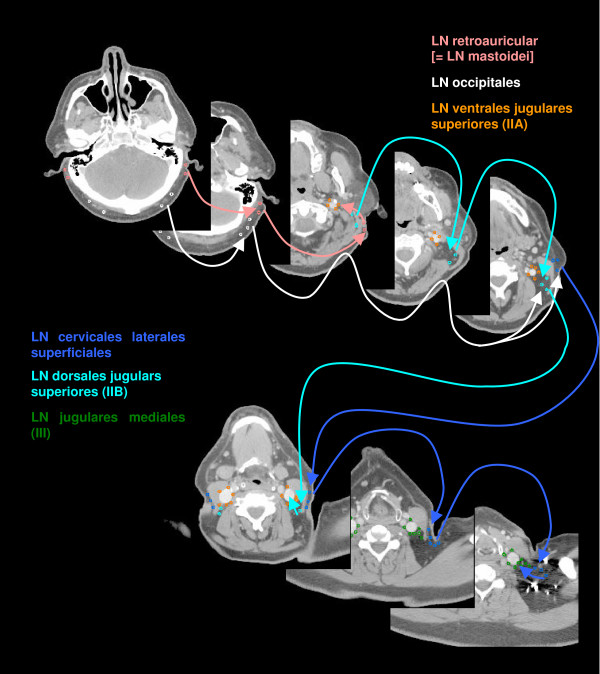
**Lymph regions and drain contoured in transversal CT slices: LN occipitales (white), LN retroauriculares [ = LN mastoidei] (pink), LN cervicales laterales superficiales (medium blue) and LN dorsales jugulares superiores (cyan) [1.8 cm slice thickness]**.

**Figure 5:**
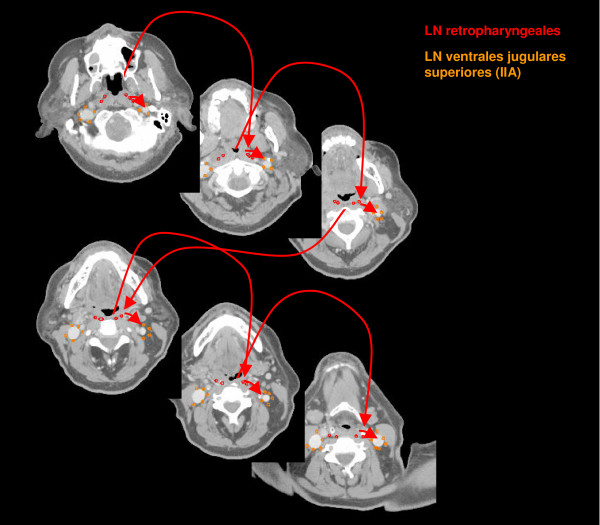
**Lymph regions and drain contoured in transversal CT slices: LN retropharyngeales (red) [1 cm slice thickness]**.

**Figure 6:**
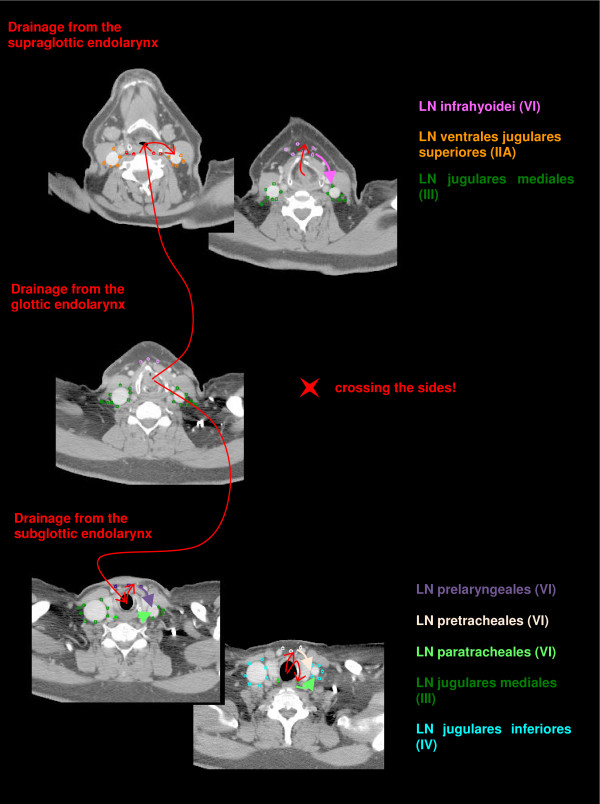
**Lymph drainage from the endolarynx contoured in transversal CT slices (red arrows) to the LN infrahyoidei (pink), LN prelaryngeales (violet), LN pretracheales (light pink) and LN paratracheales (light green)**.

**Figure 7:**
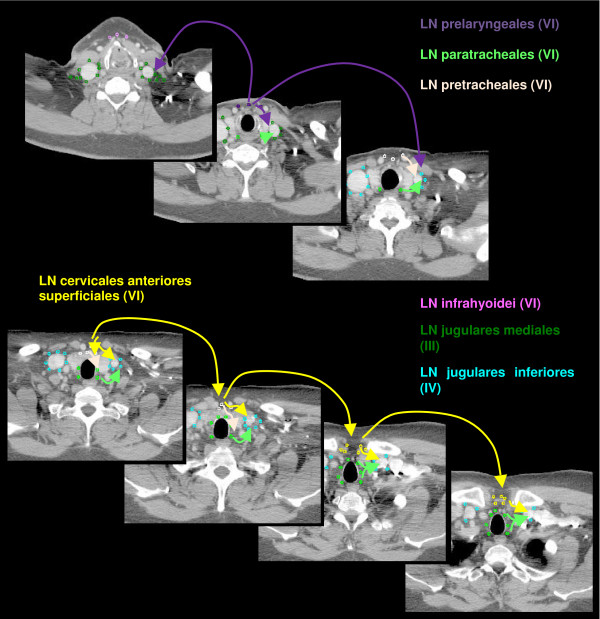
**Lymph regions and drain contoured in transversal CT slices: LN cervicales anteriores superficiales (yellow) and LN cervicales anteriores profundi subdivided into LN infrahyoidei (pink), LN prelaryngeales (violet), LN pretracheales (light pink) and LN paratracheales (light green) [1 cm slice thickness]**.

The anatomic patterns of lymphatic drainage for different body regions to their nodal stations were taken from Richter and Feyerabend *Normal lymph node topography *[12] and confirmed with other anatomy textbooks [5,13-15]. The elective irradiation of N0 patients can produce results equivalent to those obtained by neck dissection. Hence we used histopathologic analyses to create our suggested guidelines [16]. The main lymphatic routes for different organs, which are relevant in radiotherapy of the head and neck region, are summarized in Table 2. A general description of the anatomic lymph node drain for different lymph node regions can be found in Table 3 and Figure 2, 3, 4, 5, 6, 7, 8. The lymph node regions are classified into lymph node level (Table 4) adapted to Som et al. [17]. Guidelines for lymphatic CTV delineation of the most frequently cases of the different tumor entities were generated and summarized in Table 5,6,7,8.

**Table 2:** Anatomy - lymph node regions

anatomic region	organ	subregion	1. lymph node region	figure	2. lymph node region
nasal cavity	nose	anterior parts of the mucosa	LN submandibulares	3	LN ventrales jugulares superiores
		
		posterior part of mucosa	LN retropharyngeales	5	LN ventrales jugulares superiores

oral cavity	oral cavity	buccal mucosa, outer part of alveolar ridge	LN submandibulares	3	LN ventrales jugulares superiores
		
		inner part of alveolar ridge	LN submandibulares	3	LN ventrales jugulares superiores
		hard and soft palate	LN retropharyngeales	5	LN ventrales jugulares superiores
			***(crossing the sides!)***	3	
		
		gingiva of the front teeth of mandible	LN submandibulares	3	LN ventrales jugulares superiores
			LN submentales	3	LN ventrales jugulares sup./LN submand.
		
		upper gingiva	LN submandibulares	3	LN ventrales jugulares superiores
			LN retropharyngeales	5	LN ventrales jugulares superiores
			***(crossing the sides!)***	3	
		
		other gingiva of mandible	LN submandibulares	3	LN ventrales jugulares superiores
		
		Teeth	LN submandibulares	3	LN ventrales jugulares superiores
		
		floor of the mouth	LN submandibulares	3	LN ventrales jugulares superiores
			LN submentales	3	LN ventrales jugulares sup./LN submand
	
tongue		tip of tongue	LN submentales	3	LN ventrales jugulares sup./LN submand.
		
		lateral part of tongue	LN submandibulares	3	LN ventrales jugulares superiores
		
		central and posterior part of tongue	LN ventrales jugulares superiores	3	
			LN jugulares mediales	3	
		
		all	***(crossing the sides!)***		

nasopharynx			LN retropharyngeales	5	LN ventrales jugulares superiores
			LN ventrales jugulares superiores	5	
			***(crossing the sides!)***		

oropharynx		dorsal part of the oropharynx	LN retropharyngeales	5	LN ventrales jugulares superiores
			LN ventrales jugulares superiores	5	
		
		other parts	LN submandibulares	3	LN ventrales jugulares superiores
			LN ventrales jugulares superiores	3	

hypopharynx			LN jugulares mediales		
			LN paratracheales	7	LN jugulares mediales and inferiores
			LN retropharyngeales (caudal part)	5	LN ventrales jugulares superiores

larynx		supraglottic region	LN ventrales jugulares superiores	6	
			LN infrahyoidei	6	LN jugulares mediales
		
		glottic region	supraglottic region	6	
			subglottic region	6	
		
		subglottic region	LN prelaryngeales	6	LN jugulares mediales
			LN pretracheal	7	LN jugulares mediales and inferiors
			LN paratracheales	7	LN jugulares mediales and inferiores
		
		posterior part of larynx	LN paratracheales	7	LN jugulares mediales and inferiores
		
		all	***crossing the sides! no crossing*** ***between supraglottic and glottic region***		

ear		external auditory canal	LN parotidei profundi	2	LN ventrales jugulares superiores
		
		tympanic cavity	LN parotidei profundi	2	LN ventrales jugulares superiores
			LN retropharyngeales	5	LN ventrales jugulares superiores
		
		eustachian tube	LN retropharyngeales	5	LN ventrales jugulares superiores

orbit		cornea, sclera, lens, retina	---		
		
		conjunctiva	circumferentially around cornea [circulus lymphaticus]		
		
		lateral part of conjunctiva	LN parotidei profundi	2	LN ventrales jugulares superiores
			LN parotidei superficiales	2	LN ventrales jugulares superiores
		
		medial part of conjunctiva	LN faciales	3	LN submand.
			LN submandibulares	3	LN ventrales jugulares superiores

paranasal sinuses			LN ventrales jugulares superiores		
			LN retropharyngeales	5	LN ventrales jugulares superiores

cellulae mastoidei			LN retroauricular [ = LN mastoidei]	4	LN ventrales jugulares superiores

submandibular gland			LN submandibulares	3	LN ventrales jugulares superiores
			LN ventrales jugulares superiores		

parotid gland		cranial part	LN parotidei superficiales	2	LN ventrales jugulares superiores
			LN parotidei profundi	2	LN ventrales jugulares superiores
		
		caudal part	LN parotidei superficiales	2	LN ventrales jugulares superiores
			LN parotidei profundi	2	LN ventrales jugulares superiores
			LN cervicales laterales superficiales	4	LN cerv. prof. lat. mediales

thyroid gland		medial superior part	LN pretracheal	7	LN cerv. prof. lat. mediales and inferiores
		
		lateral superior part	LN jugulares mediales	7	
		
		medial inferior part	LN pretracheal	7	LN cerv. prof. lat. mediales and inferiors
			LN paratracheal	7	LN cerv. prof. lat. mediales and inferiores
			LN thyroidei		
		
		lateral inferior part	LN jugulares inferiores	7	

skin	scalp	forehead	LN parotidei superficiales	2	LN ventrales jugulares superiores
			LN submandibulares	3	LN ventrales jugulares superiores
			LN faciales	3	LN submand.
		
		temple	LN parotidei superficiales	2	LN ventrales jugulares superiores
		
		region around the mastoid process	LN retroauricular [ = LN mastoidei]	4	LN ventrales jugulares superiores
		
		parietal part of the scalp	LN retroauricular [ = LN mastoidei]	4	LN ventrales jugulares superiores
		
		occipital scalp	LN occipitales	4	LN dorsales jugulares superiores
	
	neck	nape	LN cervicales laterales superficiales	4	LN jugulares mediales
		side of the neck	LN cervicales posteriores profundi	8	LN supraclaviculares
		
		ventral part of neck	LN cervicales anteriores superficiales	7	LN pretracheal
					LN paratracheales
					LN jugulares inferiores
		
		skin over sternocleidomastoid muscle, supraclavicular, suprahyoidal, infrahyoidal region	LN jugulares		
	
	face	lateral eyelid	LN parotidei superficiales	2	LN ventrales jugulares superiores
			LN parotidei profundi	2	LN ventrales jugulares superiores
		
		medial eyelid	LN submandibulares	3	LN ventrales jugulares superiores
			LN faciales	3	LN submand.
		
		lacrimal gland	LN parotidei profundi	2	LN ventrales jugulares superiores
		cheek	LN submandibulares	3	LN ventrales jugulares superiores
		
		lower lip	LN submentales	3	LN ventrales jugulares sup./LN submand
		chin	LN submandibulares	3	LN ventrales jugulares superiores
			***(crossing the sides!)***		
		
		upper lip	LN submandibulares	3	LN ventrales jugulares superiores
	
	nose	root of the nose	LN parotidei profundi	2	LN ventrales jugulares superiores
		
		other parts of the nose	LN submandibulares	3	LN ventrales jugulares superiores
			LN faciales	3	LN submand.
	
	ear	anterior part	LN parotidei superficiales	2	LN ventrales jugulares superiores
		
		lower part	LN cervicales laterales superficiales	4	LN jugulares mediales
		posterior part	LN retroauricular [ = LN mastoidei]	4	LN ventrales jugulares superiores

**Figure 8:**
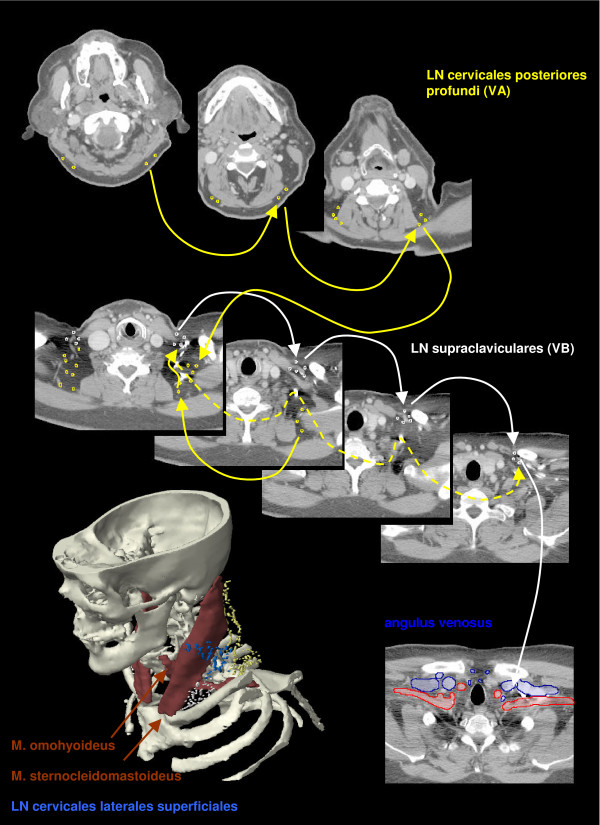
**Lymph regions and drain contoured in transversal CT slices: LN cervicales posteriores profundi (yellow) and LN supraclaviculares (white)**.

**Table 3:** Anatomy - lymph node drain

Lymph node regions	Subgroups	Anatomic site	Influx	Efflux	Figure
LN parotidei profundi	LN preauriculares	ventral of the auricle	external auditory canal	(partially over the LN parotidei superficiales)	2
	LN intraparotidei	medial of the parotid gland	tympanic cavity		
	LN infraparotidei	dorsocaudal of the parotid gland	parotid gland	to the LN ventrales jugulares superiores	
			skin of the root of the nose, the cheek, the lateral part of the eyelid and conjunctiva		

LN parotidei superficiales		on the fascia parotidea	skin of the anterior part of the ear, the forehead, the temple, the lateral part of the eyelid and conjunctiva	LN ventrales jugulares superiores	2

LN retroauriculares ( = LN mastoidei)		lateral of the mastoid process	skin of the posterior part of the ear, the region around the mastoid process, parietal part of the scalp and from the cellulae mastoideae.	LN ventrales jugulares superiores	4

LN occipitales		at the linea nuchae superior	skin at the occipital scalp	LN dorsal jugulares superiores	4
				LN cervicales laterales superficiales	

LN submentales		ventral between the two venter of the musculus digastricus	tip of tongue	LN submandibulares	3
			floor of the mouth	LN ventrales jugulares superiores	
			laterals of the two front teeth of the mandible		
			skin of the lower lip and chin		

LN submandibulares		adjacent to the submandibular gland	anterior part of the nasal cavity	LN ventrales jugulares superiores	3
			skin/mucosa of the lips/cheek, palate, teeth, gingiva, lateral tongue and floor of the mouth		
			skin from the forehead, nose and the medial part of the eyelid and the conjunctiva over inconstant LN faciales (LN buccales)		

LN facials (inconstant)		arranged around the V. angularis	skin from the forehead, nose and the medial part of the eyelid and the conjunctiva	LN submandibulares	3

LN dorsales jugulares superiores		medial of the musculus sternocleidomastoideus and dorsal of the jugular vein	LN occipitales	LN ventrales jugulares superiores	4

LN cervicales laterales superficiales		along the external jugular vein, lateral of the musculus sternocleidomastoideus	lower part of the parotid gland	LN jugulares mediales	4
			skin of the caudal part of the ear, the nape and lateral neck		

LN retropharyngeales		in the space bounded anteriorly by the pharyngeal constrictors and posteriorly by the prevertebral Fascia, cranially by the base of the skull and caudally to the os hyoideum **	nasopharynx	from cranial to caudal up to the level of the os hyoideum or to the lateral side into the LN ventrales jugulares superiores	5
			dorsal part of the oropharynx		
			soft palate		
			eustachian tube		
			tympanic cavity		
			dorsal part of the nasal cavity		

LN cervicales anteriores profundi	LN infrahyoidei	located on the membrane hyoidea	cranial half of the larynx	LN jugulares mediales	6-7
	LN prelaryngeales	on the ligamentum cricothyroideum	caudal half of the larynx		
	LN pretracheales	at the veins thyroideae inferiors	caudal half of the larynx	LN jugulares mediales and inferiores	6-7
	LN paratracheales	ventral/laterodorsal of the trachea	thyroid gland		
	LN thyroidei	at the thyroidea	thyroid gland	LN jugulares mediales and inferiores	

LN cervicales anteriores superficiales		around the vein jugularis anteriores	ventral skin of the neck	LN pre- or paratracheales	7
				LN jugulares inferiores	

LN cervicales posteriores profundi		in the neck region caudal of the LN occipitales	neck region	LN supraclaviculares	8

LN supraclaviculares		between the M. omohyoideus and the clavicular	caudal neck	sometimes over the venous jugulo-subclavian confluent or the thoracic duct on the left side and the lymphatic duct on the right side, to the angulus venosus [13,14].	8
			pharynx region		
			trachea		
			esophagus		
			LN mediastinales anteriores		
			LN axillares profundi		

**We defined the retropharyngeal level analogically to Grégoire et al. [7] and Feng et al. [27].

**Table 4:** Lymph node level (adapted to [17])

Lymph node level	Terminology	Lymph node regions	Figure
level IA	submental	LN submentales	3

level IB	submandibular	LN submandibulares	3

level IIA	ventral upper jugular group	LN ventrales jugulares superiores	2-6

level IIB	dorsal upper jugular group	LN dorsales jugulares superiores	4

level III	mediales jugular group	LN jugulares mediales	3-4, 6-7

level IV	lower jugular group	LN jugulares inferiores	6

level VA	posterior triangle group	LN cervicales posteriors profundi	8

level VB	posterior triangle group	LN supraclaviculares	8

level VI	anterior compartment	LN cervicales anteriores superficiales	6
		LN cervicales anteriores profundi:	7
		- LN infrahyoidales	
		- LN prelaryngeales	
		- LN pretracheales	
		- LN paratracheales	
		- LN thyroidei	

level retropharyngeal	retropharyngeal	LN retropharyngeales	5

level parotidal	parotidal	LN parotidei superficiales	2
		LN parotidei profundi	

level retroauricular	retroauricular	LN retroauriculares	4

level occipital	occipital	LN occipitales	4

level buccal	buccal	LN faciales	3

level external jugular	external jugular	LN cervicales laterales superficiales	4

**Table 5:** Suggested guidelines for the treatment of the neck of patients with squamous cell carcinoma of the oral cavity or oropharynx

	Oral cavity cN0						Oropharynx cN0			
	**(ventral) tongue**	**floor of tue mouth**	**hard palate**	**upper gingiva**	**lower gingiva**	**buccal mucosa**	**base of tongue**	**tonsillar fossa**	**soft palate**	**pharnygeal wall (dorsal)**

**submental (IA)**	b	i		i	i					
**submandibular (IB)**	b	i	b	b	i	i		i	i	
**ventral jugular sup. (IIA)**	b	i	b	b	i	i	b	i	b	b
**dorsal jugular sup. (IIB)**							b	i	b	b
**jugular medial (III)**	b	i	b	b	i	i	b	i	b	b
**jugular inferior (IV)**	b						b	i	b	b

**cerv. post. prof. (VA)**										

**supraclavicular (VB)**										
**retropharyngeal**			b	b					b	b

	**Oral cavity cN+**						**Oropharynx cN+**			
	**(ventral) tongue**	**floor of the mouth**	**hard palate**	**upper gingiva**	**lower gingiva**	**buccal mucosa**	**base of tongue**	**tonsillar fossa**	**soft palate**	**pharnygeal wall (dorsal)**

**submental (IA)**	b	i	b	b	i	i				
**submandibular (IB)**	b	i	b	b	i	i		i	i	i
**ventral jugular sup. (IIA)**	b	i	b	b	i	i	b	b	b	b
**dorsal jugular sup. (IIB)**							b	b	b	b
**jugular medial (III)**	b	i	b	b	i	i	b	b	b	b
**jugular inferior (IV)**	b	i	b	b	i	b	b	b	b	b

**cerv. post. prof. (VA)**	b	i	b	b	i	b	b	b	b	b
**supraclavicular (VB)**	b	i	b	b	i	b				

**retropharyngeal**								b	b	b

**Table 6:** Suggested guidelines for the treatment of the neck of patients with squamous cell carcinoma of the hypopharynx, larynx or nasopharynx

	Hypopharynx cN0			Larynx cN0				Nasopharynx cN0
	pyriform sinus	pharyngeal wall	*esophageal extension*	supraglottic	glottic	subglottic	posterior part	
**submental (IA)**								
**submandibular (IB)**								
**ventral jugular sup. (IIA)**	b	b	b	b	b	i	i	b
**dorsal jugular sup. (IIB)**								b
**jugular medial (III)**	b	b	b	b	b	b	b	b
**jugular inferior (IV)**	b	b	b	b	b	b	b	b

**cerv. post. prof. (VA)**			b					b
**supraclavicular (VB)**			b					b
**infrahyoidal (VI)**		i	i	b	b			
**prelaryngeal (VI)**		i	i		b	b		
**pretracheal (VI)**		i	i		b	b		
**paratracheal (VI)**	i	i	i		b	b	b	

**retropharyngeal**		b	b					b

**faciales**								
**parotidal**								

	**Hypopharynx cN+**			**Larynx cN+**				**Nasopharynx cN+**
	**pyriform sinus**	**pharyngeal wall**	***esophageal extension***	**supraglottic**	**glottic**	**subglottic**	**posterior part**	

**submental (IA)**		b						
**submandibular (IB)**		b						b
**ventral jugular sup. (IIA)**	b	b	b	b	b	i	b	b
**dorsal jugular sup. (IIB)**								b
**jugular medial (III)**	b	b	b	b	b	b	b	b
**jugular inferior (IV)**	b	b	b	b	b	b	b	b

**cerv. post. prof. (VA)**	b	b	b		b	b	b	b
**supraclavicular (VB)**	b	b	b		b	b	b	b
**infrahyoidal (VI)**	i	i	i	b	b			
**prelaryngeal (VI)**	i	i	i		b	b		
**pretracheal (VI)**	i	i	i		b	b		
**paratracheal (VI)**	i	i	i		b	b	b	

**retropharyngeal**	b	b	b					b

**faciales**								b
**parotidal**								b

**Table 7:** Suggested guidelines for the treatment of the neck of patients with squamous cell carcinoma in the head and neck region

	Ear			Nasal cavity		Thyroid gland			
	external auditory canal	tympanic cavity	eustachian tube	anterior part of mucosa	posterior part of mucosa	medial superior part	lateral superior part	medial inferior part	lateral inferior part
**submental (IA)**									
**submandibular (IB)**				i					
**ventral jugular sup. (IIA)**	i	b	b	i	b	b	i	b	i
**dorsal jugular sup. (IIB)**									
**jugular medial (III)**	i	b	b	i	b	b	i	b	i
**jugular inferior (IV)**						b	i	b	i

**cerv. post. prof. (VA)**	i	b	b						
**supraclavicular (VB)**									
**pretracheal (VI)**						b		b	
**paratracheal (VI)**								b	
**thyroidei (VI)**						b	b	b	b

**retropharyngeal**		b	b		b				
**faciales**									
**parotidal**	i	i							
**external jugular**									
**retroauricular**									

	**Orbit**			**Parotid gland**		**Paranasal sinus**	**Cellulae mastoideae**	**Submandibular gland**	
	**cornea,sclera,lens,retina**	**lateral part of conjunctiva**	**medial part of conjunctiva**	**cranial part**	**caudal part**				

**submental (IA)**									
**submandibular (IB)**			i	i	i			i	
**ventral jugular sup. (IIA)**		i	i	i	i	b	b	i	
**dorsal jugular sup. (IIB)**				i	i				
**jugular medial (III)**		i	i	i	i	b	b	i	
**jugular inferior (IV)**				i	i	i		i	

**cerv. post. prof. (VA)**				i	i				
**supraclavicular (VB)**				i	i				
**pretracheal (VI)**									
**paratracheal (VI)**									
**thyroidei (VI)**									

**retropharyngeal**						b	b		

**faciales**			i						
**parotidal**		i		i	i				
**external jugular**					i				
**retroauricular**							i		

**Table 8:** Suggested guidelines for the treatment of the neck of patients with carcinomas of the skin

	Skin of scalp					Skin of neck			Skin of nose	
	forehead	temple	mastoid region	parietal part of scalp	occipital scalp	nape, side of neck	ventral part of neck	supraclavi-cular region	root of the nose	other parts
**submental (IA)**										
**submandibular (IB)**	i									i
**ventral jugular sup. (IIA)**	i	i	i	i	i	i		i	i	i
**dorsal jugular sup. (IIB)**					i					
**jugular medial (III)**	i	i	i	i	i	i	b	i	i	i
**jugular inferior (IV)**						i	b	i		

**cerv. post. prof. (VA)**						i				
**supraclavicular (VB)**						i				
**cerv. ant. superf. (VI)**							b			
**pretracheal (VI)**							b			
**paratracheal (VI)**							b			

**faciales**	i									i
**parotidal**	i	i							i	
**external jugular**					i					
**retroauricular**			i	i						
**occipital**					i					

	**Skin of face**					**Skin of ear**				
	**lateral eyelid**	**medial eyelid**	**lacrimal gland, cheek**	**lower lip, chin**	**upper lip**	**anterior part**	**lower part**	**posterior part**		

**submental (IA)**				b						
**submandibular (IB)**		i	i	b	i					
**ventral jugular sup. (IIA)**	i	i	i	b	i	i	i	i		
**dorsal jugular sup. (IIB)**										
**jugular medial (III)**	i	i	i	b	i	i	i	i		
**jugular inferior (IV)**							i			

**cerv. post. prof. (VA)**										
**supraclavicular (VB)**										
**cerv. ant. superf. (VI)**										
**pretracheal (VI)**										
**paratracheal (VI)**										

**faciales**		i								
**parotidal**	i		i			i				
**external jugular**							i			
**retroauricular**								i		
**occipital**										

## Lymph node level

**Figure 9:**
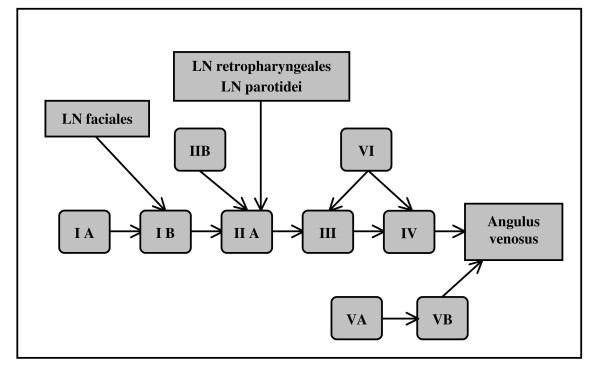
**Schematic scheme of main direction of lymph node flow in the head and neck region**.

**Figure 10:**
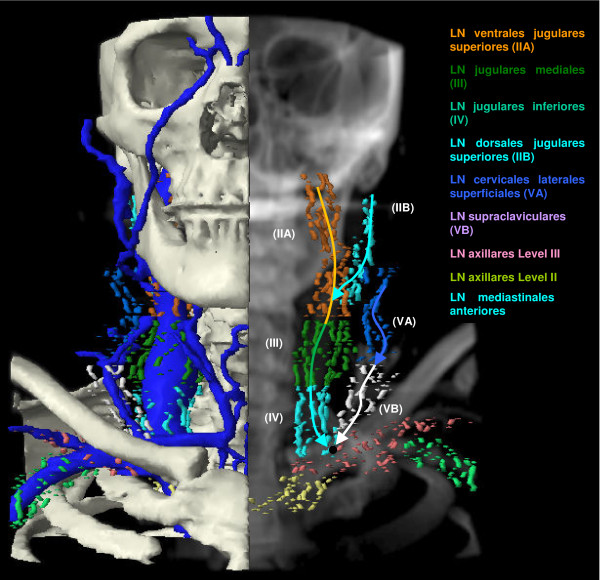
**Coronar DRR with different lymph node regions, bones and veins**. The black circle symbolises the angulus venosus.

The main lymph node groups are classified analogically to Som et al. [17] into different levels (Table 4). The level IA contains the submental LN and the level IB the submandibular LN. The LN jugulares ( = LN cervicales laterales profundi) are subdivided in four groups - the LN ventrales jugulares superiores (level IIA), the LN dorsales jugulars superiores (level IIB), LN jugulares mediales (level III) and LN jugulares inferiores (level IV) (Figure 9, 10). We included the retrostyloid space, which range cranial to the scull base, analogically to Som et al. [17] in level IIA. There are only few data available about NM in the retrostyloid space, because a neck dissection do not extend beyond the posterior belly of digastric muscle [7]. Gregoire et al. 2006 [10] recommend to include the retrostyloid space in the CTV for nasopharyngeal cancer or NM in the caudal level II. For N0 patients there are not enough clinical data available to exclude this space from the CTV. The LN level IIB are localised dorsal of the LN level IIA, with the LN level IIA are near to the jugular vein and the LN level IIB are not attached to the jugular vein [17]. The caudal limit of the level IV is set to the clavicle [17]. The level V is divided into the LN cervicales posteriores profundi (level VA) cranial of the musculus omohyoideus and the LN supraclaviculares (level VB) (Figure 8) [18,19]. The definition of "level V" varies much in the literature. For this reason we decided to follow a definition based on anatomic lymph node regions combined with the surgical and histopathological information, which follows mostly the definition of Rotterdam [1,3,4,9,20,21]. The anterior compartment between the both levels III and IV is called level VI and includes the LN cervicales anteriores superficiales and profundi. The main lymph drain flows from level II over level III and IV over the truncus lymphaticus jugularis and/or subclavius to the angulus venosus of the same side of the body (Figure 9, 10) [4]. The truncus can end directly in a vein or on the right side over a ductus lymphaticus dexter or on the left side over the truncus thoracicus. The lymph form level IA flows over level IB to level IIA and the lymph from level VA over level VB to the angulus venosus. Level VI drains to level III and IV. There are still more lymph node regions, which are not respected by the classification by Robbins et al. [19]. The parotidal level contains the LN parotidei superficiales and profundi and drain to level IIA just as well as the level retropharyngeal and level retroauricular, which contains the LN retropharyngeales and LN retroauriculares, respectively. The LN faciales are classified into the level buccales, which drain to the level IB. The level external jugular includes the LN cervicales laterales superficiales and has efflux to the level III.

## General selection and delineation of the lymphatic CTV

The spread of head and neck tumors into cervical LN is rather consistent and follows predictable pathways, with increasing risk at each level, if the adjoining proximal level is involved [2]. The incidence of occult metastases in LN ranges between 20% and 50% and NM in cN+ (metastatic involvement of LN via clinical assessment) patients ranges between 35% and 80% for all tumors of the oral cavity, pharyngeal and laryngeal tumors, except glottic tumors (0-15% occult metastases). This indicates the necessity to include the adjacent lymph node regions in the CTV.

Most parts of the head and neck region has rich lymph node vessels. But some sites, as the true vocal cord, the paranasal sinuses and the mediales ear, have only few or no lymphatic vessels at all [7]. Typically the lymph drain remains on one body side. Only some structures, like the soft palate, the base of tongue and the larynx have crossing lymph drain [7]. The retropharyngeal lymph vessel, which involving for example the lymph from the posterior pharyngeal wall and the nasopharynx, often cross the side.

The lymph drainage from the endolarynx takes different ways (Figure 6, Table 2). The supraglottic endolarynx drains through the membrana thyrohyoidea directly to the LN ventrales jugulares superiores (level IIA) or to the LN infrahyoidei and continuing to the LN jugulares mediales (level III). The lymph from the subglottic endolarynx flows through the ligamentum cricothyroideum to the LN prelaryngeales, LN pretracheales and LN paratracheales and further to the more caudal located LN lower jugulars (level IV). The glottis region of the endolarynx has only few lymph vessels, which are connected mostly to the upper endolarynx, but also to the lower endolarynx [6,12-14].

The distribution of pathologic confirmed NM depends on three major points - the clinical evaluation of the lymph node sides, the primary tumor side and tumor size [7].

• Patients with cN+ have a much higher incidence of NM than patients with cN0 (no metastatic involvement of LN via clinical assessment) [22]. Gregoire et al. [7] summarised the results from the Head and Neck Service at Memorial Sloan-Kettering Cancer Center between 1965 and 1989 with 33% metastatic diseases in prophylactic neck dissections and 82% in therapeutic neck dissections. In patients, who underwent therapeutic neck dissection, the pattern of metastatic nodes was similar to the one observed in cN0 patients with one extra level of NM [7].

• Tumors of different anatomic locations in the head and neck region drain in different percentage to different lymph node level. In cN+ patients Gregoire et al. 2000 described an incidence of metastatic disease in LN is highest in patients with nasopharyngeal cancer (80%) and lowest in patients with tumors of the oral cavity (36%). Patients with a laryngeal cancer have a much higher incidence of NM (54%) in contrast to cancer of the oral cavity, hypopharynx or oropharynx (17-25%), if they have a T3-T4 stage tumor. And more cranial and anterior localised tumors mainly drain into the level I to III in contrast to more caudally located tumors, which mainly drain into level II to V. Nasopharyngeal and oropharyngeal tumors drain not only to the level IIA but also to the level IIB (Table 5, 6). Tumors of the oral cavity, hypopharyngeal and laryngeal tumors are mainly associated to the level IIA and less to the level IIB [7].

• The incidence of metastatic lymph node involvement increases with the primary tumor size [7,22,23].

• More factors, which influence the lymph node invasion, are the tumor differentiation, kertinization status, lymphatic vessel invasion in the tumor specimen, and whether other lymph node levels are involved [2]. Remmert et al. [22] found for example 16.7% NM for G1 tumors, 36.5% for G2 and 58.9% for G3.

• If the tumor crosses the midline bilateral treatment of the LN is necessary [24].

The CTV of the lymph node regions should encompass all regions, who have a probability to contain NM of 10% or more [2,7]. If the NM infiltrates adjacent structures, the inclusion of this structure and the associated lymph drain in the CTV must individually be assessed [10].

Summarizing the highest incidence for over all NM can be found in patients with cN+, a laryngeal cancer stage T3/4 and/or nasopharyngeal cancer (cN+). Patients with tumors of the oral cavity (even cN+ or T3/4) have the lowest incidence for NM [7].

Clinical and pathologic neck node distributions support the concept, that not all lymph node level has to be treated for squamous cell tumors of the head and neck region [7]. All concepts base on retrospective data with possible bias because of mostly selected patients. Some surgery techniques for the neck dissection do not perform lymph node dissection in all level, e.g. level IIb is often not examined, and will result in an underestimation of the involvement of these lymph node levels [7]. Another point is that the incidence of NM in retropharyngeal and paratracheal LN can only be estimated clinically. Medial retropharyngeal LN has been reported to be very rarely involved by radiologic analysis in contrast to the lateral retropharyngeal LN [25,26]. Therefore it seems to be adequate only to define the lateral retropharyngeal LN as target [27]. To exclude all these problems would require large multicenter randomized trials.

Both sides of the neck exhibit a similar pattern of node distribution, but with a lower incidence in the contralateral neck. There are only few data on the pattern of contralateral NM.

This must be assessed by recalculation of relative involvement probabilities to the subregions. The results are still more based on clinical judgment rather than from scientific evidence. Recalculated from the analysis of Gregoire et al. 2000 [7] more than 90% of all NM are found on the ipsilateral side for tumors in the oral cavity or hypopharynx. Tumors of the oropharynx or larynx spread to the contralateral side in 11-14% of the patients. Only for tumors in the nasopharynx over 40% of the contralateral LN show metastases. The relative number of contralateral metastases must be correlated with the absolute number of pathologic LN per bilateral level to find the incidence per neck side. If the tumor invades the midline, the lymph drain to both sides of the neck and therefore both sides should be included in the CTV. Some anatomic regions have crossing lymph node drainage, like the soft palate, the tongue, the larynx and the nasopharynx [12]. But even for those tumors contralateral involvement occurs at a much lower frequency than on the ipsilateral side [7], but should also be included in the CTV (Table 4, 5, 6). As well is the incidence of retropharyngeal LN higher in cN+ patients, in whom involvement of other neck node levels was also documented [7].

Infiltration of level V is very rare, except level IV is involved or more than a single lymph node in level I-III has metastatic disease (Table 4, 5, 6) [7,20]. Chone et al. [28] detected NM of level VA in pN0 patients with a prevalence of 2.3% and in pN+ patients with 16.7%. The prevalence was highest for tumors of the pharynx (23.1%) in contrast to tumors of the oral cavity with 3.6%. No NM were found for other tumor sides and there are no isolated metastases in level VA [28].

Timon at al. [3] found in patients with advanced cancer of the larynx, hypopharynx or cervical esophagus NM in 20% and 43% of the patients, respectively. 10% of the patients had positive paratracheal NM alone in a histopathological negative cervical neck dissection. In subglottic cancer the incidence of paratracheal NM can be up to 50% [7]. Therefore the LN paratracheal should be included in the CTV for patients with advanced laryngeal or hypopharyngeal tumors or extension of the tumor to the cervical esophagus.

Metastases are labelled as 'skip metastases', if the lymph node involvement bypass a lymph node level and involve the next but one level. Skip metastases are very rare [20]. Remmert et al. [22] analysed 405 patients with head and neck carcinoma and found no skip metastases. A series of the Head and Neck Service at Memorial Sloan-Kettering Cancer Center found skip metastases in 2.5% of the cN0 patients [7,23]. Only an analysis of tumors of the oral tongue by Byers et al. [29] reached a rate of 12% skip metastases in the level IIb, III and IV.

## Squamous cell cancer of the oral cavity

The oral cavity itself has primary lymph drainage to the LN submandibulares and submentales (Table 1). Tumors of the tongue drain also directly to level IIA and III [6,30]. The lymph from the hard palate and upper gingiva flows additionally to the LN retropharyngeales. Squamous cells tumors of the oral cavity have the lowest absolute incidence of NM of all head and neck regions, but the overall incidence of NM for N+ patients is still high with more than 30% [7,22,24,31]. In level IIA, II and III the relative incidence of NM is higher than 10%, independent of the tumor location [6,7]. These levels should be included in the lymphatic CTV (Table 5). The general probability for contralateral NM is low with < 10% [6]. But the lymph drainage of the tongue has direct significant cross-over with a higher risk of contralateral NM [6,7,32]. And the hard palate and upper gingiva have additional lymph drain to the LN retropharyngeales of both sides [7]. Yen et al. [33] found for patients with squamous cell carcinoma of the buccal mucosa a high incidence of NM in the ipsilateral level I to III. Patients with this tumor entity showed only 2% contralateral NM, why it is reasonable only to treat the ipsilateral side [16,33].

Robbins et al. [18] has suggested that for N0 patients no elective lymph node dissection of level IIB is necessary [24]. Analogically the level IIB may not be included in the CTV for patients with N0. Byers et al. [29] found a high incidence of NM in level IV in patients with tumors of the (ventral) tongue, which should therefore be included in the CTV, even for N0 patients [24]. Shah et al. [23] described a prevalence of NM in level IV of 3% in patients underwent elective node dissection and 17% in patients with therapeutic neck dissection, wherefore this level should be included in the CTV for all N+ patients of the oral cavity. Oral cavity carcinoma in cN0 patients nearly never metastasizes to level V, which therefore may not be included in the CTV [24]. The incidence for NM in the parotidal LN in patients with oral squamous cell carcinoma is very rare (2.5%) with about 75% intraglandular NM [34].

## Squamous cell cancer of the oropharynx

The overall incidence of NM is over 60% for squamous cell tumors of the oropharynx [6,7,30]. The primary drainage of the tongue base is to level II and III of both sides [6]. An analysis of tumors of the oral tongue by Byers et al. [29] reached a rate of 12% skip metastases in the level IV, for which reason this level should be included in the lymphatic CTV (Table 5). The lymphatic drainage of the tonsil is mainly to level IIA, without crossing the sides [6,12,30]. Certainly histopathologic evaluation showed level I (and also level V) involvement only in association with involvement of other levels (N+ disease) [20]. Tumors of the soft palate and dorsal pharyngeal wall show NM on both sides via crossing lymph vessels [6,7,12]. For these tumors the retropharyngeal LN should be included in the CTV [7,35].

## Squamous cell cancer of the hypopharynx

The lymphatic drain of the piriform sinus is to the LN paratracheales and directly to the level III [6,12,30]. The pharyngeal wall has additional efflux to the LN retropharyngeal and the ipsilateral LN prelaryngeales, pretracheales and infrahyoidei. The overall incidence for NM in patients with tumors of the hypopharynx is very high with over 70% [7,22]. The number of reported detected contralateral NM is low (<10%), but should not be neglected because of the anatomic cross-lymphatic drainage of the hypopharyngeal region [6]. The LN cervicales anteriores profundi (Figure 6, 7), included in level VI, drain lymph not only from the hypopharynx, larynx and thyroid gland, but also from the cervical trachea and upper esophagus [7]. The incidence of pathologic LN in this region is reported rarely. Therefore in the case of tumor infiltration of the cervical trachea or the upper esophagus respectively the level VI should be included in the CTV (Table 6). All hypopharyngeal tumors has a high probability of ipsilateral paratracheal NM, for which reason this region should be included in the CTV [3,7,36]. As for patients with tumors of the oral cavity the level IIb must not be included in the CTV for patients with N0 as suggested by Robbins et al. [18]. Histopathologic evaluation showed level I involvement for tumors of the pharyngeal wall in association with involvement of other levels (N+ disease) [20]. The prevalence of level V NM is only in N+ patients high enough to encompass this region in the CTV [20]. For all tumors of the pharyngeal wall the retropharyngeal LN should be included in the CTV [7].

## Squamous cell cancer of the larynx

The lymphatic drainage of the larynx is different for the supraglottic and subglottic region (Figure 6). The supraglottic endolarynx drains to level IIA, LN infrahyoidei and level II, whereas the subglottic endolarynx drains to level VI (especially LN prelaryngeales, pretracheales, paratracheales) and level IV (Table 6). The glottic region has few lymph vessels, which drain to both regions [6,12-14,21,30]. The reported overall incidence for NM varies between 26% and 55% [7,22]. Especially the supraglottic larynx has a rich lymphatic drainage, resulting in a high incidence of occult cervical metastases [37]. The number of occult NM is about 20% [4,21]. Even the glottic region has few lymph vessels; the number of NM for advanced tumors adds up to 32% [21]. The number of NM in level II, III and IV is very high for all laryngeal cancers [4,6,22,38]. Especially supraglottic tumors are at risk for crossed lymphatic drainage. The mechanism by which this occurs is still debatable [6]. As for patients with tumors of the larynx the level IIb must not be included in the CTV for patients with N0 as suggested by Robbins et al. [18]. Laryngeal tumors has a high probability of paratracheal NM, especially tumors with subglottic extension, for which reason this region should be included in the CTV [3,7,36]. Even for N+ patients the involvement of level I is very rare and can be omitted [7,38].

## Squamous cell cancer of the nasopharynx

The lymphatic vessels drain mainly to the LN retropharyngeales, level IIA and VA. Inconsistent channels can drain to the LN parotidales [6,30]. Squamous cell tumors of the nasopharynx show a very high rate of NM in 80% of the patients [7]. Even for N0 patients the incidence of NM in the bilateral level IIA, IIB, III, IV, VA and VB is high and should be included in the lymphatic CTV (Table 6) [6,7,39]. The lymph vessels in the retropharyngeal region are often crossing the sides. Accordingly the number of contralateral NM (30%) is very high in patients with nasopharyngeal cancer [6,7,35] and should be included in the lymphatic CTV.

## Other tumors of the head and neck region

The distribution of cervical NM from primary parotidal carcinoma is rarely reported. Hence there is no consensus as to which extent the cervical level should be irradiated. Chrisholm et al. [40] found in the ipsilateral level I to V more than 20% NM each. Therefore all these level should be encompassed in the CTV (Table 7).

Squamous cell carcinomas in other location in the head and neck regions are rarely reported and all present analyses bases on small patient groups. Our suggested guidelines contribute mostly on anatomic lymph drain and medical experience. Because of the high overall incidence (<30%) of ipsilateral NM in squamous cell cancer of the maxilla, treatment of the ipsilateral neck should be considered even in cases with a negative clinical examination [41,42] (Table 7). Patients with auricular squamous cell carcinoma present NM in 10%-30% of the cases [43-45]. Clark et al. [44] found, that the parotid gland was the commonest side of node metastases, followed by LN retroauriculares and level II, III and V (Table 7). For the treatment of thyroid carcinoma the level II, II and IV show a high incidence of NM [46].

## Tumors of the skin

**Figure 11:**
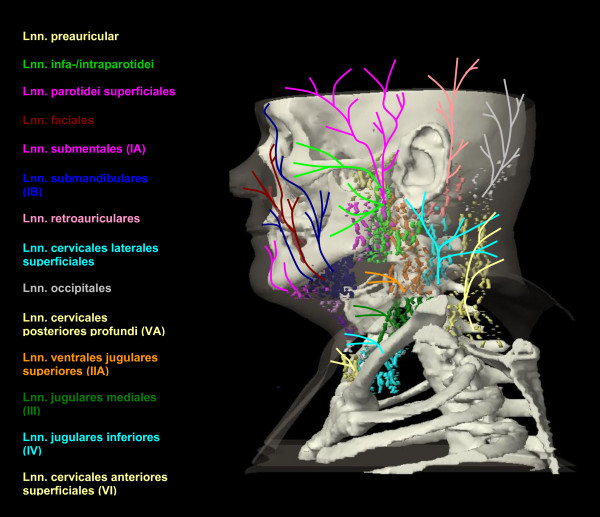
**Lymph drain from the skin outlines as a schema on a capital view (for the systematic listing see Table 2): LN preauriculares (yellow, cranial), LN infra-/intraparotidei (light green), LN parotidei superficiales (pink, cranial), LN facials (brown), LN submentales (pink, ventral), LN submandibulares (dark blue), LN retroauriculares (rose), LN cervicales laterales superficiales (cyan cranio-dorsal), LN occipitals (grey), LN cervicales posteriores profundi (yellow, dorsal), LN ventrales jugulares superiores (orange), LN jugulares mediales (dark green), LN jugulares inferiores (cyan, caudo-ventral), LN cervicales anteriores superficiales (yellow, ventral)**.

The curative treatment of basal cell carcinoma and squamous cell carcinoma with N0 status mostly includes no treatment of the lymph node regions because of insufficient evidence [47]. For patients with clinical or histological affected lymph nodes the CTV should encompass the lymph node regions listed in Table 8, but at least the affected lymph node region with one additional region. For melanomas the affected lymph node region and two additional regions should be encompassed in the lymphatic CTV. The lymph from the medial eyelid drains to the LN submandibulares and LN faciales, whereas the lymph from the lateral eyelid drains to the LN parotidei profundi and superficiales (Table 8, Figure 11) [12]. The lymph of the upper lip flows to the LN submandibulares and the lymph of the lower lip flows also to the LN submentales with possible crossing of the sides. The anterior parts of the ear drain to the preauriculares, the lower parts to the LN cervicales laterales superficiales and the posterior parts to the retroauriculares LN. The lymph vessels of the forehead and temple run to the LN parotidei superficiales and the lymph vessels of the parietal part of the scalp to the LN retroauriculares. The occipital scalp is drained by the LN occipitales. The dorsal and lateral neck regions have outflow to the LN cervicales posteriors profundi as well as to LN cervicales laterales superficiales. The lymph of the ventral parts of the neck flows to LN cervicales anteriores superficiales and forwards to the LN pre- and paratracheales. The skin of the supraclavicular region drains to LN jugulares.

## Conclusions

We have reviewed the expected lymphatic drainage of different parts of the head and neck region and correlated this with the current used level system and histopathologic experience. The results are contoured on various CT slices and summarized in Table 5, 6, 7, 8.

## Competing interests

The authors declare that they have no competing interests.

## Authors' contributions

HV carried out the literature review, performed the statistical analysis and the typing. CFH participated in the design and coordination of the analysis and the writing of the manuscript. All authors read and approved the final manuscript.
